# Supplementary material for: Development of the Nude Rabbit Model
Source: Stem Cell Reports. 2021 Feb 18;16(3):656–65. doi: 10.1016/j.stemcr.2021.01.010 (PMC7940256; doi:10.1016/j.stemcr.2021.01.010)
Supplement: Document S1. Figures S1–S5 [file mmc1.pdf]

Stem Cell Reports, Volume *16*

Supplemental Information

Development of the Nude Rabbit Model

Jun Song, Mark Hoenerhoff, Dongshan Yang, Ying Yang, Cheng Deng, Luan Wen, Linyuan Ma, Brooke Pallas, Changzhi Zhao, Yui Koike, Tomonari Koike, Patrick Lester, Bo Yang, Jifeng Zhang, Y. Eugene Chen, and Jie Xu

Title:

Development of the Nude Rabbit Model

Supplementary information

Supplementary Figure 1. Non-nude (WT and *FOXN1*$^{+/-}$) rabbits outgrew NuRabbits, beginning at eleven weeks of age. Data presented as mean ± standard error of means (SEM) from fifteen non-nude or eleven nude rabbits at each time point. Related to Figure 1.

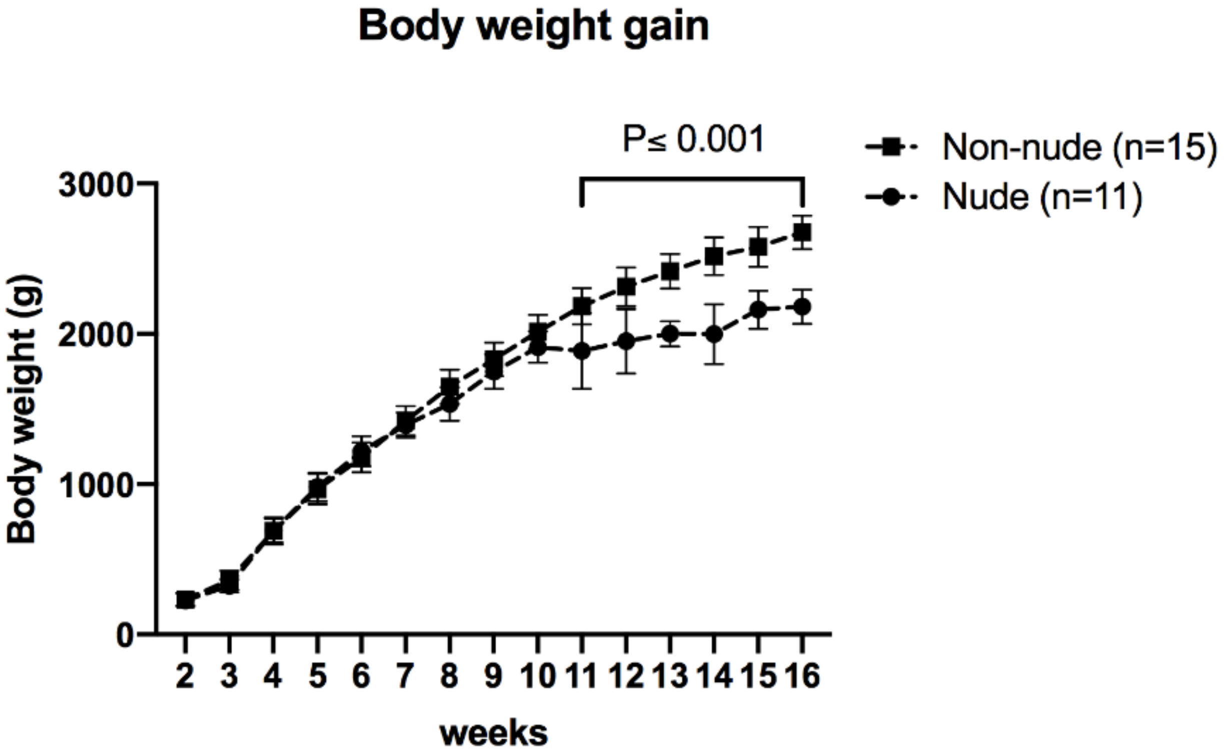

Supplementary Figure 2. Nail dystrophy in NuRabbits (right), compared to normal nail growth in a wild type rabbit (left). Related to Figure 2.

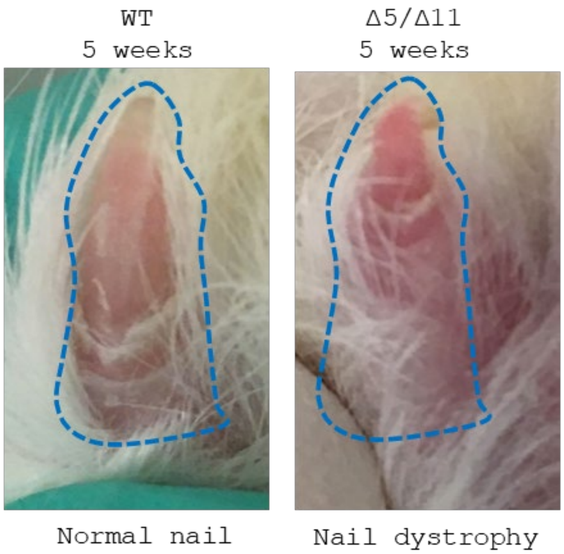

Supplementary Figure 3. Lung pathology of animal 17M049. NuRabbit 17M049 without prophylactic antibiotics developed severe bacterial bronchopneumonia. There is filling of bronchioles (arrowhead) and alveoli with neutrophils, and multifocal foci of necrosis (arrow) characterized by hypereosinophilic cellular debris and degenerative neutrophils scattered within the parenchyma. Scale bar: 50 µm. Related to Figure 3.

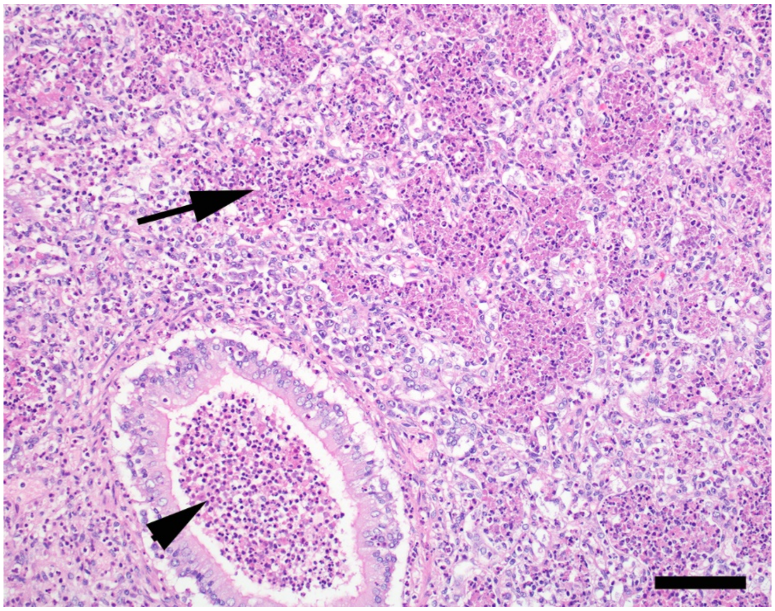

Supplementary Figure 4. Hair loss phenotype in a homozygous Δ10/Δ10 NuRabbit at different ages. Related to Figure 2.

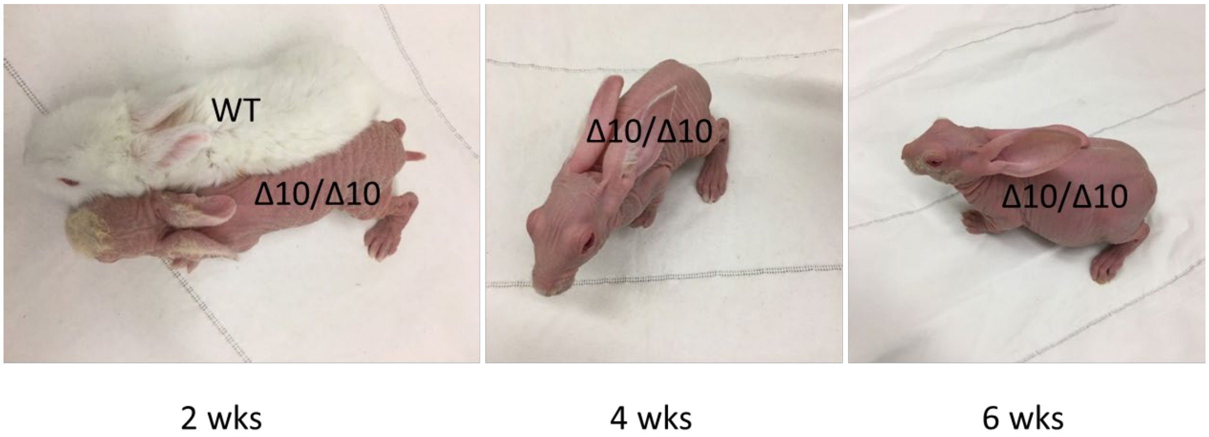

Supplementary Figure 5. Rabbit allows easy mammary gland intraductal injection. (A) Representative image of a rabbit nipple. (B) Visible mammary gland ducts after intraductal injection of PBS with 0.04% trypan blue using a 27 G needle in a rabbit. (C) The mouse nipple (within the white cycle) and a 27 G size needle. Related to Figure 4.

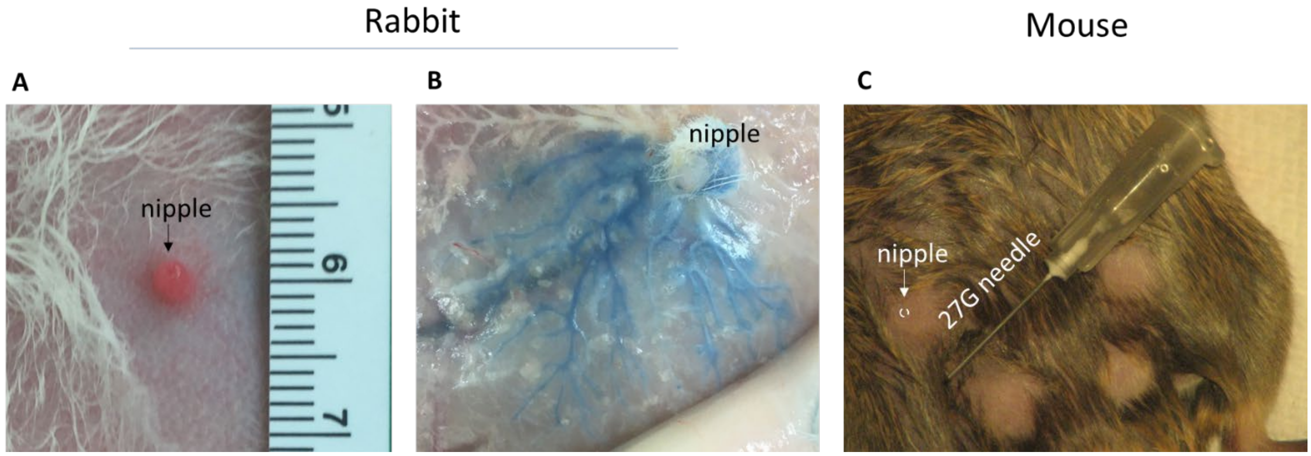

Supplementary Video 1. Restoration of blood flow in a NuRabbit after successful transplantation of a TEBV to its left common carotid artery. Related to Figure 5.
