# Supplementary material for: Development of the Nude Rabbit Model
Source: Stem Cell Reports. 2021 Feb 18;16(3):656–65. doi: 10.1016/j.stemcr.2021.01.010 (PMC7940256; doi:10.1016/j.stemcr.2021.01.010)
Supplement: Document S2. Article plus Supplemental information [file mmc3.pdf]

Stem Cell Reports
Resource
OPEN ACCESS

Development of the Nude Rabbit Model

Jun Song,[1,4] Mark Hoenerhoff,[2,4] Dongshan Yang,[1] Ying Yang,[3] Cheng Deng,[3] Luan Wen,[1] Linyuan Ma,[1] Brooke Pallas,[1,2] Changzhi Zhao,[1] Yui Koike,[1] Tomonari Koike,[1] Patrick Lester,[2] Bo Yang,[3] Jifeng Zhang,[1] Y. Eugene Chen,[1] and Jie Xu[1,*]
[1]Center for Advanced Models and Translational Sciences and Therapeutics, University of Michigan, Ann Arbor, MI 48109, USA
[2]Unit for Laboratory Animal Medicine, University of Michigan Medical School, Ann Arbor, MI, USA
[3]Department of Cardiac Surgery, University of Michigan School of Medicine, Ann Arbor, MI, USA
[4]These authors contributed equally
*Correspondence: jiex@umich.edu
https://doi.org/10.1016/j.stemcr.2021.01.010

SUMMARY

Loss-of-function mutations in the forkhead box N1 (*FOXN1*) gene lead to nude severe combined immunodeficiency, a rare inherited syndrome characterized by athymia, severe T cell immunodeficiency, congenital alopecia, and nail dystrophy. We recently produced *FOXN1* mutant nude rabbits (NuRabbits) by using CRISPR-Cas9. Here we report the establishment and maintenance of the NuRabbit colony. NuRabbits, like nude mice, are hairless, lack thymic development, and are immunodeficient. To demonstrate the functional applications of NuRabbits in biomedical research, we show that they can successfully serve as the recipient animals in xenotransplantation experiments using human induced pluripotent stem cells or tissue-engineered blood vessels. Our work presents the NuRabbit as a new member of the immunodeficient animal model family. The relatively large size and long lifespan of NuRabbits offer unique applications in regenerative medicine, cancer research, and the study of a variety of other human conditions, including immunodeficiency.

INTRODUCTION

The nude mouse (NuMouse) as an experimental animal model has been well established and applied in diverse biomedical research fields, including immunology, cancer research, stem cell therapy, and skin regeneration (Zhang et al., 2012). The nude phenotype is caused by mutation of the *FOXN1* gene, which plays a pivotal role in the differentiation of thymic and skin epithelial cells (Brissette et al., 1996; Grabowska and Wilanowski, 2017; Lee et al., 1999; Meier et al., 1999; Nowell et al., 2011; Zuklys et al., 2016). In human patients, loss-of-function mutations in the *FOXN1* gene lead to a well-described phenotype of nude combined immunodeficiency, which includes congenital alopecia, nail dystrophy, and T cell immunodeficiency (Palamaro et al., 2014; Rota and Dhalla, 2017).

One major application of NuMice is to serve as the recipient animals in allo- and xenotransplantation studies, which take advantage of their severely compromised immune response phenotype (Pantelouris, 1973). For example, in a stem cell teratoma assay, human embryonic stem cells (ESCs) or induced pluripotent stem cells (iPSCs) are often transplanted into NuMice, in order to evaluate the growth rate and germ-layer origins of the resulting teratoma. This method represents the current gold standard for the functional evaluation of ESCs and iPSCs, regarding their capacity to differentiate into a diverse array of tissue types (Brivanlou et al., 2003; Hentze et al., 2009).

We recently reported the production of immunodeficient rabbits by CRISPR-Cas9-mediated disruption of genes involved in lymphocyte development or function, such as *IL2RG*, *RAG1*, *RAG2*, *PRKDC*, and *FOXN1* (Song et al., 2017). We reasoned that an immunodeficient animal model of larger size and longer lifespan than its mouse counterpart may prove useful in many translational studies.

In the present study, we report the development and characterization of *FOXN1* mutant NuRabbits, and demonstrate their use as recipients in xenotransplantation experiments.

RESULTS

Establishment of the NuRabbit colony

Previously we produced *FOXN1* mutant rabbits by CRISPR-Cas9-mediated gene disruption (Song et al., 2017). The founder animals (F0) are mosaic, carrying wild-type (WT) alleles encoding the full-length FOXN1 protein consisting of 667 amino acids (aa) and one or more mutant alleles (Song et al., 2017). The present work is based on three different mutant alleles within the founder rabbits (Figure 1A): (1) Δ5, a deletion of 5 bp, predicted to result in a frameshift from proline at aa position 84 (P84) with a truncated size of 120 aa; (2) Δ10, a deletion of 10 bp, predicted to result in a frameshift from threonine at aa position 81 (T81) with a truncated size of 299 aa; and (3) Δ11, a deletion of 11 bp, predicted to result in a frameshift from glutamine at aa position 83 (Q83) with a truncated size of 118 aa.

To establish *FOXN1* null rabbits as a research resource, we first bred two F0 rabbits with WT partners to evaluate germline transmission ability and to produce F1-generation animals. Both founders proved to be fertile (Figure 1B), and 10

 | © 2021 The Author(s).
This is an open access article under the CC BY license (http://creativecommons.org/licenses/by/4.0/).

A

FOXN1 (complement strand)

Allele type	Sequence	Predicated protein size, type
WT	CCCTCCCTGCCCCCGCACAGCCCCAGCATTGCGTCACCGG	667 a.a., normal, NCBI#: XP_002718973.2
Δ10	CCCTCC----------ACAGCCCCAGCATTGCGTCACCGG	299 a.a., frameshifted from T81
Δ11	CCCTCCCTGCCCC-----------AGCATTGCGTCACCGG	118 a.a., frameshifted from Q83
Δ5	CCCTCCCTGCCCCCGC-----CCCAGCATTGCGTCACCGG	120 a.a., frameshifted from P841

B

Parent 1	Parent 2	No. of litters	No. of kits	Heterozygous				$FOXN1^{null}$			
				**No. of Het**	No. of Δ5/wt	No. of Δ10/wt	No. of Δ11/wt	**No. of Hom**	No. of Δ5/Δ10	No. of Δ5/Δ11	No. of Δ11/Δ11
F0 founder	WT	2	21	**10**	2	3	5	**N/A**	N/A	N/A	N/A
F1 Het	F1 Het	6	39	**19**	5	2	12	**12**	4	5	3
F2 Hom	F1 Het	3	17	**5**	0	0	5	**12**	3	0	9

Figure 1. Establishment of the NuRabbit colony

(A) *FOXN1* mutant indels that are present in the NuRabbit colony. Underlined in WT: Cas9 guide RNA targeting sequencing.
(B) Breeding summary of NuRabbits.

of a total 21 kits (47.6%) were heterozygous *FOXN1* mutants ($FOXN1^{+/-}$). Of these 10, 2 carry the Δ5, 3 carry the Δ10, and 5 carry the Δ11 mutant allele.

Next, we demonstrated that $FOXN1^{+/-}$ (i.e., wt/Δ5, wt/Δ10, or wt/Δ11) rabbits can be used to establish and maintain the NuRabbit colony. All F1-generation $FOXN1^{+/-}$ rabbits, regardless of their specific mutation type, are healthy and phenotypically indistinguishable from WT animals, and can be successfully housed in conventional animal facilities (Shayne et al., 2019). To date, we have produced six litters by interbreeding F1 $FOXN1^{+/-}$ animals (Figure 1B). A total of 39 kits were born, consisting of 8 WT (21%), 19 $FOXN1^{+/-}$ (49%), and 12 $FOXN1^{null}$ (30%), representing a near-Mendelian distribution. The 19 $FOXN1^{null}$ rabbits consist of various combinations of mutant alleles, including Δ5/Δ10 (n = 4), Δ5/Δ11 (n = 5), and Δ11/Δ11 (n = 15).

We also tested if $FOXN1^{null}$ rabbits are fertile and can be used for colony maintenance purposes. In one attempt (Figure 1B), one adult $FOXN1^{\Delta11/\Delta11}$ male was bred with three $FOXN1^{wt/\Delta11}$ females and produced 17 kits, of which 12 were $FOXN1^{\Delta11/\Delta11}$ and 5 were $FOXN1^{wt/\Delta11}$.

With orally provided prophylactic antibiotics (e.g., sulfamethoxazole-trimethoprim) and enhanced husbandry sanitation practices (Shayne et al., 2019), $FOXN1^{null}$ rabbits can live >1 year (and counting). As of May 4, 2020, we housed five adult $FOXN1^{null}$ rabbits of ages 217 (n = 1), 244 (n = 1), 330 (n = 2), and 446 (n = 1) days.

Together, this work outlines the establishment of a robust NuRabbit (i.e., $FOXN1^{null}$) colony; it demonstrates that the colony can be maintained by breeding clinically healthy and reproductively fertile $FOXN1^{+/-}$ rabbits and $FOXN1^{null}$ males, and that $FOXN1^{null}$ rabbits can live beyond 1 year of age and possibly longer under proper management.

Nude appearance of NuRabbits

Regardless of the mutation type, $FOXN1^{null}$ rabbits show the hallmark nude phenotype (Figures 2A–2C), and are hence referred to as NuRabbits. In contrast, WT and $FOXN1^{+/-}$ rabbits do not show any signs of hair loss. Such difference in phenotype made the visual identification of NuRabbits possible, even without PCR or sequencing-based genotyping assays. Among the three mutant genotypes, though, it appears that Δ5/Δ10 and Δ5/Δ11 NuRabbits show higher extents of hairless than the Δ11/Δ11 animals (Figures 2A–2C).

Histologic analysis of NuRabbit skin reveals dilated follicular ostia, which contains variable amounts of keratin debris, or irregularly formed hair shafts, accompanied by thinning of the follicular epithelium and decreased number and size of sebaceous glands within follicular adnexa (Figure 2D).

Hair growth in NuRabbits exhibited a cyclic pattern (Figure 2E). For example, in the Δ5/Δ10 NuRabbits, sparse and curly hairs grow on NuRabbits within the first 2–3 weeks of life, albeit much less than those on non-nude (i.e., WT and $FOXN1^{+/-}$) rabbits. Severe hair loss starts at approximately 4 weeks of age and peaks about 1 week later, with some

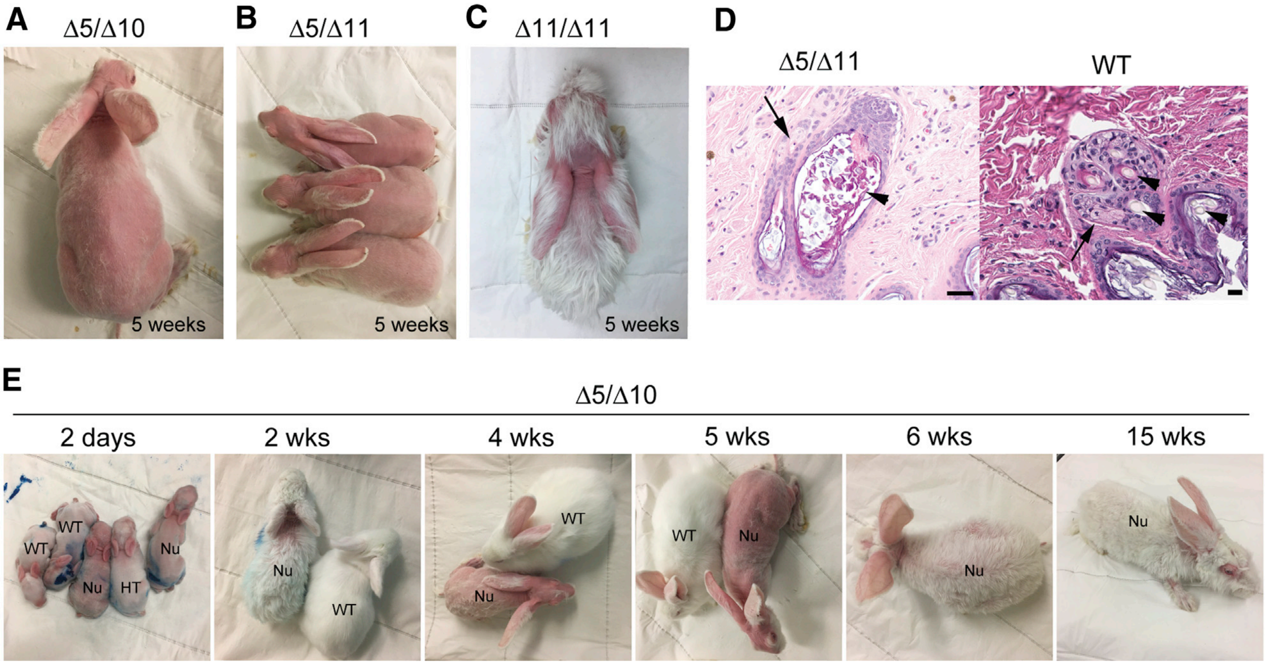

Figure 2. Nude phenotype of NuRabbits

(A–C) NuRabbits of different genotypes at 5 weeks of age. (A) Δ5/Δ10, (B) Δ5/Δ11, (C) Δ11/Δ11.

(D) Representative photomicrographs of hair follicles in a Δ5/Δ11 NuRabbit (left) and a WT rabbit (right). Sections of skin in the NuRabbit (left) are characterized by dilated follicular ostia (arrowhead) containing variable amounts of keratin debris or irregularly formed hair shafts, accompanied by thinning of the follicular epithelium and decreased number and size of sebaceous glands (arrow) within follicular adnexa, compared with haired skin of WT rabbits (right), which have well-developed sebaceous units (arrow) and multiple follicles with intact hair shafts (arrowheads) per adnexa. Bars, 20 μm.

(E) Hair patterns in Δ5/Δ10 NuRabbits at different ages. WT, wild type; HT, heterozygous *FOXN1* knockout; Nu, NuRabbit.

animals showing nearly complete hair loss. This stage is followed by hair regrowth at week 6, and eventually stabilizes as a phenotype characterized by lack of hair around the neck and upper back and a sparse hair coat on other parts of the body. NuRabbits of Δ5/Δ11 and Δ11/Δ11 genotypes exhibit similar cyclic hair loss/growth patterns (data not shown).

Other phenotypes of NuRabbits include diffuse nail dystrophy and a slightly lower body weight over time compared with their WT littermates (Figures S1 and S2).

NuRabbits are immunodeficient

Lymphoid tissue defects

On postmortem examination, there is a marked reduction or absence of lymphoid tissues in NuRabbits. The thymus is often unidentifiable grossly (Figure 3A), and other lymphoid tissues (i.e., spleen and lymph nodes) are also markedly reduced in size. Histologically, an absence or marked decrease in lymphoid tissue is the primary phenotypic alteration (Figure 3B). In contrast to WT animals, in which thymic cortical and medullary zones are present in appropriate thickness with adequate numbers of cortical lymphocytes, the thymus in the NuRabbit is generally devoid of all lymphoid elements, with only remnants of medullary thymocytes and few scattered mononuclear cells and granulocytes within a loose connective tissue stroma. Similarly, in the spleen, in contrast to WT animals in which the white and red pulp is adequately represented, with dense zones of T lymphocytes within periarteriolar lymphoid sheath (PALS) areas and multiple variably sized follicles of B lymphocytes, the spleen in NuRabbits is markedly reduced in size and there is a paucity of all lymphoid elements (Figure 3B) and absence of PALS and lymphoid follicles, with very few small individual clusters of lymphocytes and scattered granulocytes remaining.

In other organs with resident lymphoid tissue, there is a general decrease in these populations regardless of the organ. There is a general absence of bronchial-associated lymphoid tissue in the lung and reduction of lymphocytes in the gastrointestinal-associated lymphoid tissue in the stomach and intestines. There is a generalized reduction in lymphocyte numbers in the peripheral lymph nodes and decreased lymphocytes in the appendix (Figure 3B) and sacculus rotundus, with evidence of lymphocytolysis

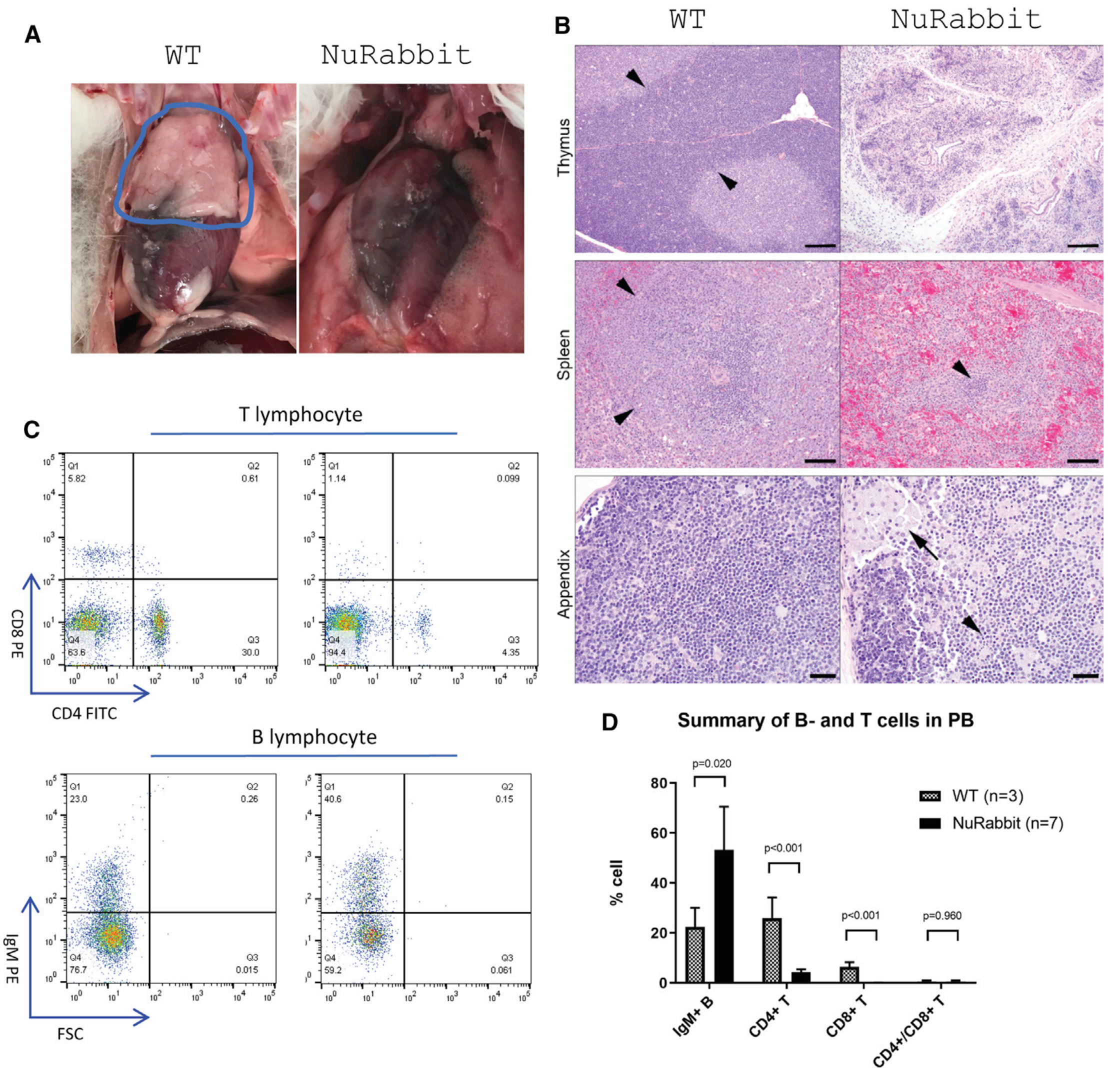

Figure 3. NuRabbits are immunodeficient

(A) Lack of thymus development in a NuRabbit (right), compared with normal thymus development in a WT rabbit (circled in blue).

(B) Sections of thymus (bar, 100 μm), spleen (bar, 50 μm), and appendix (bar, 20 μm) from WT rabbits (left) compared with NuRabbits (right). Compared with thick cortical lymphoid areas (arrowheads) in the thymus of WT rabbits, NuRabbits show marked reduction in lymphoid elements, with only a few clusters of cells remaining in loose connective tissue stroma of thymic lobules. The spleen of WT rabbits has large lymphoid follicles (arrowheads), compared with a general paucity of lymphoid tissue in NuRabbits, with only small remnants remaining (arrowhead). In sections of the appendix, WT rabbits have dense sheets of lymphoid tissue within the lamina propria, compared with a patchy loss of lymphocytes in NuRabbits, accompanied by pyknotic cellular debris (arrowhead) and large foamy macrophages phagocytosing degenerate lymphocytes (arrow).

(C) Representative flow cytometry results of peripheral blood lymphocytes in WT and NuRabbits.

(D) Summary of B and T cell populations in peripheral blood from seven NuRabbits in comparison with that from three WT rabbits.

characterized by pyknosis of lymphocytes and phagocytosis of cellular debris by large foci of activated macrophages containing foamy cytoplasm and pyknotic cellular debris.

T cell deficiency in peripheral blood of NuRabbits

Peripheral blood was collected from the central ear artery of NuRabbits and analyzed for populations of circulating lymphocytes. By flow cytometry, there was a significant decrease in the percentage of T lymphocytes in NuRabbits compared with WT rabbits. With regard to T cell subsets, populations of CD8-positive cells were close to eliminated, and CD4-positive T cells were significantly decreased from 30% to 4% (Figures 3C and 3D). However, B lymphocytes remained within normal reference ranges.

NuRabbits are prone to lung infections

Without prophylactic antibiotics, NuRabbits (n = 5) universally developed symptoms and signs of respiratory tract infection and ultimately succumbed to respiratory failure. In one NuRabbit (case 17M049) that was euthanized due to moribund status, postmortem examination revealed bronchopneumonia that was characterized histologically by multifocal consolidation of the pulmonary parenchyma and filling of airways with neutrophils, macrophages, eosinophilic proteinaceous material, and cellular debris (Figure S3), consistent with bacterial bronchopneumonia. PCR testing (Charles River Laboratories, Research Animal Diagnostic Services, Wilmington, DE) confirmed the presence of *Bordatella bronchiseptica* within affected lung sections.

These findings are consistent with an immunodeficient phenotype in NuRabbits, which echoes the observations in NuMice, nude rats, and patients, and supports the use of NuRabbits as a model animal for the study of nude severe combined immunodeficiency (SCID) (Gallo et al., 2017; Rolstad, 2001; Szadvari et al., 2016).

NuRabbits support the growth of teratoma derived from human iPSCs

In addition to their use as disease models, nude animals are often used in xenograft studies utilizing human stem cells or tumor cells, taking advantage of their compromised immune rejection responses. Here we worked to demonstrate that NuRabbits support xenotransplantation-based experiments in a teratoma assay using human iPSCs (Figure 4A).

Seven animals of two different age groups were utilized. At the time of iPSC implantation, group A (n = 3) NuRabbits (NuRabbits-A) were 7–9 weeks of age, and group B (n = 4) NuRabbits (NuRabbits-B) were 2–3 weeks of age. Each NuRabbit received intramuscular injections of 1×10^6 iPSCs in each hindleg. In the control group, NuMice (n = 2) at 6 weeks of age were used, also receiving 1×10^6 iPSCs in each hindleg. At the endpoint of the experiment, tumors were removed following euthanasia, and the lengths of the longest axis (Dmax) and the shortest axis (Dmin) of each teratoma were measured as an indicator of teratoma size.

In group A, teratomas grew in all four injection sites in two animals, but did not grow in two injection sites (4/6, 67%) of the third animal (Figure 4B). In contrast, teratomas grew in eight of eight injection sites (100%) in group B animals, indicating that NuRabbits in the 2–3 weeks age range may serve as better recipients for iPSC teratoma growth than those in the 7–9 weeks age range (Figure 4B). This success rate is similar to that in the control NuMice group, where teratomas grew in all four injection sites in two animals (100%) (Figures 4B and 4C).

All teratomas isolated from NuRabbits were next subjected to germ-layer analysis (Figure 4C). The results show that the injected iPSCs are competent to support the development of all three germ layers (i.e., endoderm, mesoderm, and ectoderm), similar to the findings in the positive control NuMice group (Figure 4D).

In the control NuMice group, teratomas reached a Dmax value of 2.1 ± 0.2 cm 8–12 weeks post-implantation (p.i.) (Figure 4E). The size of the teratoma (∼2 cm Dmax) and the duration of the assay (8–12 weeks p.i.) are commonly reported when NuMice are used for iPSC teratoma assays (Cooke et al., 2006; Gordeeva and Nikonova, 2013). Teratomas from group A NuRabbits were of dimensions similar to those from NuMice (Figure 4E). Interestingly, however, group B NuRabbits supported a much faster teratoma growth course, evidenced by the larger Dmax value (4.2 ± 0.5 cm) being attained in a shorter amount of time (6–9 weeks p.i.), compared with the NuMice group (2.1 ± 0.2 cm in 8–12 weeks). Similarly, the Dmin value was also much larger in group B NuRabbits (3.1 ± 0.4 cm) than in the control NuMice (1.7 ± 0.1 cm).

Together, these data show that NuRabbits can serve as useful recipients for the iPSC teratoma growth assay. In comparison to the gold standard NuMice protocol, NuRabbits at 2–3 weeks age supported larger teratoma growth (6–8 times larger in volume) in a shorter duration (on average 3 weeks shorter).

Transplantation of tissue-engineered blood vessel to a NuRabbit

We next conducted a transplantation experiment using patient-specific stem cell-derived tissue-engineered blood vessels (TEBVs) in a Δ5/Δ10 NuRabbit. Human iPSC-derived smooth muscle cells (SMCs) were used to generate TEBVs (Figure 5A). The diameter of a TEBV is in the range of 2–3 mm, similar to that of a rabbit common carotid artery (CCA), but much larger than that of a mouse CCA, which is approximately 0.5 mm (Figures 5B and 5C). The small size of a mouse and consequently the small sizes of its blood vessels are inhibitory for a TEBV transplantation procedure. In contrast, the size of an adult NuRabbit is similar to that of a human infant and the size of its CCA allows practice transplantation. Indeed, through a surgical procedure, TEBV was successfully transplanted to the left CAA (Figure 5D) and blood flow was restored (Video S1) in a 6-month-old NuRabbit.

This TEBV transplantation experiment demonstrates the size advantage of NuRabbits over immunodeficient mice.

DISCUSSION

In the present study, we established a colony of NuRabbits, characterized their phenotype, and validated their use in xenotransplantation experiments. Similar to *Foxn1* null mice and rats, NuRabbits are hairless, are athymic, and

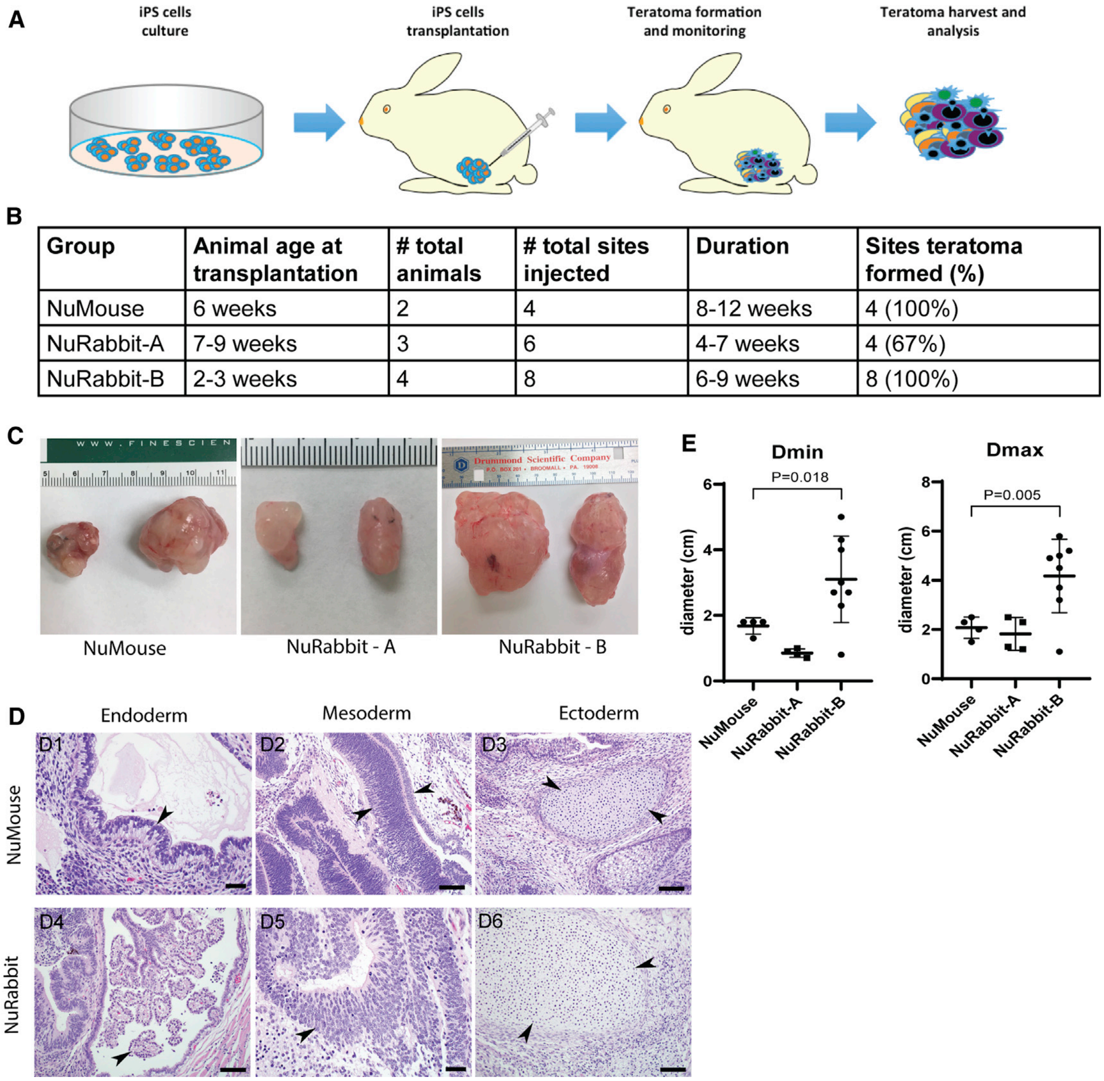

Group	Animal age at transplantation	# total animals	# total sites injected	Duration	Sites teratoma formed (%)
NuMouse	6 weeks	2	4	8-12 weeks	4 (100%)
NuRabbit-A	7-9 weeks	3	6	4-7 weeks	4 (67%)
NuRabbit-B	2-3 weeks	4	8	6-9 weeks	8 (100%)

Figure 4. NuRabbits support iPSC-derived teratoma growth
(A) Illustration of teratoma test in NuRabbits.
(B) Summary of teratoma formation in NuRabbits.
(C) Representative pictures of teratomas generated in NuRabbits and NuMice.
(D) Germ-layer staining of teratomas obtained from NuMice and NuRabbits. Arrowheads point to endoderm-derived epithelium (D1 and D4), mesoderm-derived nervous system tissues (D2 and D5), and ectoderm-derived cartilage foci (D3 and D6). Scale bars, 20 μm in D1; 50 μm in D2, D3, and D4; 20 μm in D5; and 50 μm in D6.
(E) Summary of teratoma sizes in NuRabbits and NuMice.

exhibit nail dystrophy (Rolstad, 2001; Szadvari et al., 2016). Importantly, NuRabbits are immunodeficient, characterized predominantly by lymphopenia, similar to that observed in *Foxn1* null mice and rats (Rolstad, 2001; Szadvari et al., 2016). Without prophylactic antibiotics, these animals universally succumb to lung infections following weaning. However, by housing NuRabbits within a positively pressured flexible film bubble (bioBubble, Fort Collins, CO), with HEPA-filtered air and enhanced husbandry sanitation protocols (e.g., autoclaved food, hay, and water), and the use of antibiotics (Shayne et al., 2019), NuRabbits can survive well beyond 1 year of age. Although we did not test if homozygous female NuRabbits are fertile, in an effort to reduce infection risk, male NuRabbits are proven to be fertile. Heterozygous *FOXN1* knockout rabbits, both female and male, are healthy and fertile and have been used to maintain the colony. As of this writing, we have maintained the NuRabbit line for more than 3 years. These results show that NuRabbits are established and can be maintained as a breeding line for research purposes.

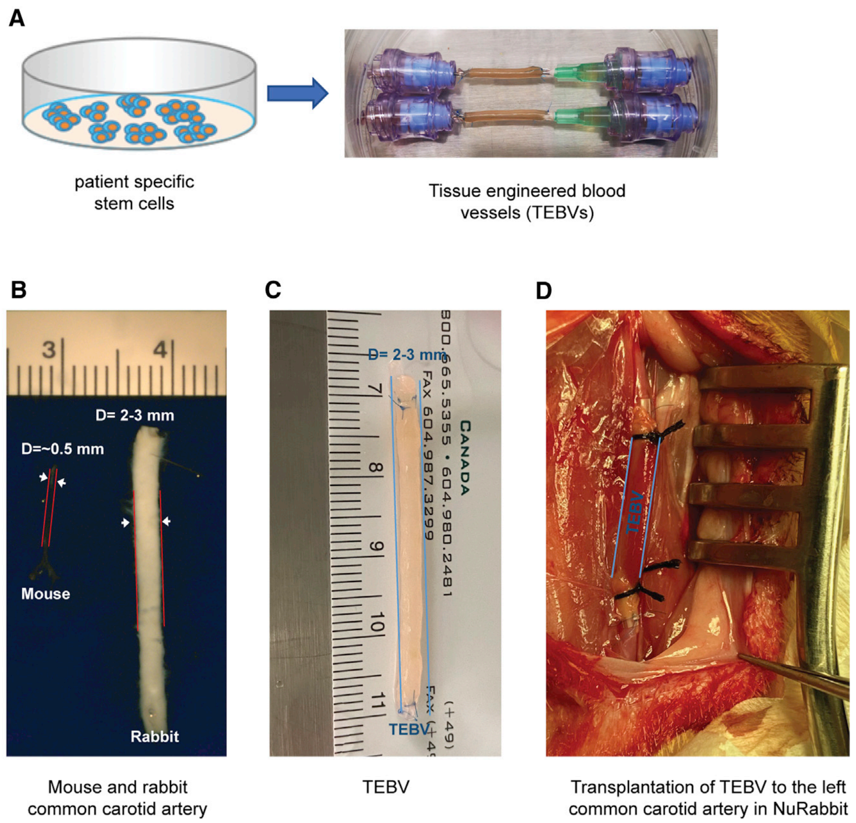

Figure 5. Transplantation of TEBV to NuRabbit

(A) Illustration of patient-specific stem cell-derived TEBVs.

(B) Representative images of common carotid arteries from an adult mouse (left) and an adult rabbit (right). D, diameter.

(C) A representative TEBV.

(D) Representative image of a TEBV after transplantation to the left common carotid artery in a NuRabbit.

In the present work, however, many NuRabbits carry compound heterozygous mutations. It is necessary to continue the breeding practice to establish a larger colony of NuRabbits that carry homozygous mutant alleles. Of the three mutant allele types, we are currently focusing on the Δ10 mutation, as we have observed a prominent "nude" phenotype in one homozygous Δ10/Δ10 NuRabbit (Figure S4), and are continuing breeding to confirm. It is of interest to note that NuRabbits of different mutation types manifest variable extents of hair loss in the present work, which corroborates the findings in a recent report (Du et al., 2019) where mice carrying different *Foxn1* mutations showed different hair-loss phenotypes, although they all had thymic aplasia and were immunodeficient. Future studies are needed to elucidate the molecular mechanisms of how different *FOXN1* mutations affect hair and thymus development in rabbits.

We anticipate that NuRabbits will contribute to translational biomedical research immediately in several areas. First, NuRabbits can serve as a model system to facilitate the development of novel therapeutics for nude SCID. For example, since rabbits are much larger than rodents, it is possible to harvest large quantities (>10^6–10^7) of bone marrow or peripheral blood cells from an adult NuRabbit that typically weighs 2–3 kg. These cells can be cultured *in vitro* and genetically corrected using emerging gene editing tools such as CRISPR-Cas9, followed by autologous transplantation back to the same NuRabbit.

Second, NuRabbits can be used as a large-sized model system for cell-line-derived xenograft (CDX) and patient-derived xenograft (PDX) studies, which are widely used in cancer research and drug development. Currently, NuMice are the predominant model species for CDX/PDX studies. However, tumor growth is restricted in a smaller host such as a mouse, whereas in a larger host, such as a rabbit, tumors may be propagated and grown for longer periods of time to larger sizes, to facilitate longer-term enhanced study of xenograft tumors. A NuRabbit-based CDX/PDX model, under proper animal welfare guidance, will provide an unprecedented tool to study large tumors. The large size of a NuRabbit would also open new possibilities for otherwise very difficult orthotypic CDX/PDX transplantations. For example, mammary gland intraductal transplantation, which is a useful technique for breast cancer research, could be much more easily conducted in a rabbit than in a mouse (Figure S5).

Third, NuRabbits may find broad applications in regenerative medicine. In the present work, we demonstrated the use of NuRabbits as recipients for TEBVs. Patient-specific stem cell-derived TEBVs are considered a promising

therapy for blood vessel diseases such as aneurysms. The safety and efficacy of TEBVs need to be thoroughly evaluated in preclinical animal models before they proceed to clinical trials. Here we have shown that, while TEBV transplantation is extremely challenging to conduct in mice, the procedure can be easily performed in NuRabbits. Another example application in regenerative medicine is long-term safety and efficacy studies of stem cell transplantation therapies, for which it is desirable to follow up over the span of years rather than months. In this application, NuRabbits would be a preferable choice of model over immunodeficient mice.

In summary, we have established and maintained a colony of NuRabbits; characterized their phenotype as hairless, athymic, and immunodeficient; and validated their use in stem cell-based xenograft experiments. This new animal model has the potential for a wide variety of applications in translational biomedical research.

EXPERIMENTAL PROCEDURES

Animals

The animal maintenance, care, and use procedures were reviewed and approved by the Institutional Animal Care and Use Committee of the University of Michigan, an AAALAC International fully accredited institution. All procedures were carried out in accordance with the approved guidelines. The nude rabbits were housed in a bioBubble within a standard specific-pathogen-free facility with enhanced sanitary care (Shayne et al., 2019). For breeding the nude rabbits, WT New Zealand White rabbits were obtained from Covance or Charles River. WT rabbits from various commercial vendors may be positive for *B. bronchiseptica*, which generally does not cause clinical signs of respiratory disease in immunocompetent rabbits. As a result, WT rabbits should be tested for the presence of *B. bronchiseptica* and other pathogens prior to introduction into a NuRabbit colony.

Analysis of lymphocytes in the peripheral blood

Approximately 1 mL of peripheral blood was collected from the central ear artery of NuRabbits into an EDTA-coated tube. The red blood cells were lysed using RBC Lysis Buffer (00-4300-54, eBioscience) as instructed by the manufacturer, and antibody incubation was performed as described in our previous work (Song et al., 2017). Briefly, single cells were stained with fluorescein isothiocyanate-conjugated anti-rabbit CD4 (KEN-4, Bio-Rad), anti-rabbit CD8 (12.C7, Bio-Rad), and anti-rabbit IgM (NRBM, Bio-Rad) for 30 min at 4°C, avoiding direct light, and then washed with 1 mL cold PBS containing 1% FBS at 300*g*, for 5 min. The resuspended cells were stained with the secondary antibody, phycoerythrin-conjugated rat anti-mouse IgG1 (12-4015-82, eBioscience), for another 30 min at 4°C, avoiding direct light. After being washed again, the red blood cells were resuspended in 300 μL of cold PBS containing 1% FBS. The suspended cells were analyzed by flow cytometry using a MoFlo Astrios cell sorter (Beckman). The fluorescence-activated cell sorting data were analyzed using FlowJo software v.10 (Tree Star, Ashland, OR, USA).

Teratoma formation test in NuRabbits

The human iPSC line DYP0250 (ACS-1004) was purchased from ATCC, Inc. Cells were cultured in mTeSR1 (85850, STEMCELL Technologies) medium in a 10-cm Matrigel (354277, Corning)-coated culture plate. Cells were dissociated using ReleSR (05872, STEMCELL Technologies) with careful attention to avoid dissociating human iPSCs into single cells. The dissociation medium was gently aspirated and culture medium was added, in order to detach the colonies from the plate. The cell suspension was then collected and centrifuged for 5 min at 300*g*, 4°C, and the cells were resuspended in growth factor-reduced Matrigel, at 1×10^6 cells/50 μL, and slowly loaded into an insulin syringe and immediately placed on ice.

NuRabbits or NuMice were anesthetized using vaporized isoflurane in oxygen at 5% for induction and 2%–3% for maintenance. Anesthetized animals were placed in sternal recumbency, and the skin at the injection sites on both femurs was disinfected, and the cell suspension was slowly injected intramuscularly. After full recovery from anesthesia, the animals were monitored for up to 12 weeks after transplantation, or until the teratoma diameter reached 2 cm for mice or 4 cm for rabbits, in any dimension. At this point, the animal was humanely euthanized, according to university-approved procedures, and the teratoma aseptically collected and fixed for *in vitro* assay.

Transplantation of tissue-engineered blood vessels

TEBVs were a gift from Dr. Bo Yang's laboratory. TEBVs were derived from human iPSC-derived SMCs using a procedure previously described (Gong et al., 2020). To set up an *in vivo* animal model, we transplanted the TEBVs as an interposition graft, into the carotid artery of a NuRabbit. An adult NuRabbit was anesthetized (10 mg/kg ketamine + 0.5 mg/kg diazepam i.v., with additional inhalation of isoflurane 1%–3%). Once the anesthesia took effect, a 3- to 5-cm median cervical incision was made. The left CCA was carefully exposed and replaced by TEBV as an interposition graft with end-end cuffing (Cho and Song, 2018). In detail, after heparin was administered intravenously, the left CCA was clamped proximally and distally 3–4 cm apart. The left CCA was then divided in the middle between the clamps. An end-to-end anastomosis was made between the left CCA and the TEBV segments. The end-to-end rejoining was made with a polyvinyl tube. The proximal part of the left carotid artery was inserted into the tube, and the intimal side of the artery was everted and ligated using a 2-0 thin thread. Finally, the cuff-mounted carotid artery was inserted into one end of the TEBV. The same procedure was performed at the other end of the TEBV, with the distal carotid artery. The TEBV was de-aired before the graft was tied. The degree of anastomosis potency was confirmed by visualizing the pulsate bright red blood flow in the transplanted TEBV after the vascular clamps were released. After confirmation of blood flow and hemostasis, the wound area was closed.

Mammary gland intraductal injection

The rabbit was anesthetized with isoflurane, and adequate anesthetic depth was confirmed by the absence of hindlimb reflexes after foot pinching stimulation. A 1-mL Luer-lock syringe was loaded with 100 μL PBS containing 0.04% trypan blue. The teat was gently

held with the thumb and index finger and lifted for the intraductal injection. While the lifted position of the teat was maintained, a 27G blunt-tip needle was carefully cannulated into a mammary duct of interest. After cannulation, the Luer-lock syringe was gently twisted on the hub of the blunt-tip infusion needle until it was locked in place, followed by injection of the solution. To visualize trypan blue distribution, the animal was humanely euthanized, after which the skin was removed to expose the mammary gland.

Necropsy and histopathology

Animals were humanely euthanized. Representative samples of brain, heart, lung, kidney, spleen, thymus, stomach, small intestine (duodenum, jejunum, ileum), large intestine (colon, cecum, rectum), sacculus rotundus and appendix, lymph nodes (submandibular, mesenteric), salivary gland, pancreas, adrenal gland, skeletal muscle (biceps femoris), tongue, and testis and epididymis were collected and fixed in 10% neutral buffered formalin for 48 h and then transferred to 70% ethanol and processed for histopathology. Histology processing was performed by the In Vivo Animal Core histology laboratory within the Unit for Laboratory Animal Medicine. Briefly, formalin-fixed tissues were processed through graded alcohols and cleared with xylene, followed by infiltration with molten paraffin using an automated VIP5 or VIP6 tissue processor (Tissue-Tek, Sakura-Americas, Torrance, CA). Following paraffin embedding using a Histostar embedding station (Thermo Fisher Scientific, Hanover Park, IL), tissues were then sectioned on an HM 355S rotary microtome (Thermo Fisher Scientific, Hanover Park, IL) at 4 μm thickness and mounted on glass slides. Following deparaffinization and hydration with xylene and graded alcohols, formalin-fixed, paraffin-embedded slides were stained with Harris hematoxylin (Thermo Fisher Scientific, Cat. No. 842), differentiated with Clarifier (Thermo Fisher Scientific, Cat. No. 7401), blued with bluing reagent (Thermo Fisher Scientific, Cat. No. 7301), stained with eosin Y, alcoholic (Thermo Fisher Scientific, Cat. No. 832), and then dehydrated and cleared through graded alcohols and xylene and coverslipped with Micromount (Leica Cat. No. 3801731, Buffalo Grove, IL) using a Leica CV5030 automatic coverslipper. Slides were examined with an Olympus BX43 light microscope and images acquired with an Olympus DP26 digital microscopy camera. Slides were examined by a board-certified veterinary pathologist (M.H.).

Statistical analysis

Data are presented as either the mean ± SEM or individual data points. Statistically significant differences between groups were determined using the Student t test. Differences were considered statistically significant if $p < 0.05$.

SUPPLEMENTAL INFORMATION

Supplemental information can be found online at https://doi.org/10.1016/j.stemcr.2021.01.010.

AUTHOR CONTRIBUTIONS

J.X. conceived the idea. J.S., B.Y., E.Y.C., and J.X. designed experiments. J.S., M.H., D.Y., Y.Y., C.D., L.W., L.M., B.P., C.Z., Y.K., T.K., P.L., J.Z., and J.X. conducted experiments and analyzed the data. J.S., M.H., B.P., E.Y.C., and J.X. wrote the manuscript.

ACKNOWLEDGMENTS

We would like to thank the In Vivo Animal Core for their necropsy and histology expertise. This project is supported by the National Institutes of Health (AI122510 to J.X., HL147527 and HL129778 to Y.E.C., and HL141891 and HL151776 to B.Y.).

Received: May 15, 2020
Revised: January 18, 2021
Accepted: January 19, 2021
Published: February 18, 2021

REFERENCES

Brissette, J.L., Li, J., Kamimura, J., Lee, D., and Dotto, G.P. (1996). The product of the mouse nude locus Whn, regulates the balance between epithelial cell growth and differentiation. Genes Dev. *10*, 2212–2221.

Brivanlou, A.H., Gage, F.H., Jaenisch, R., Jessell, T., Melton, D., and Rossant, J. (2003). Stem cells. Setting standards for human embryonic stem cells. Science *300*, 913–916.

Cho, S., and Song, I.H. (2018). Cuff technique for small-diameter vascular grafts in the systemic arterial circulation of the rat. Korean J. Thorac. Cardiovasc. Surg. *51*, 423–426.

Cooke, M.J., Stojkovic, M., and Przyborski, S.A. (2006). Growth of teratomas derived from human pluripotent stem cells is influenced by the graft site. Stem Cells Dev. *15*, 254–259.

Du, Q., Huynh, L.K., Coskun, F., Molina, E., King, M.A., Raj, P., Khan, S., Dozmorov, I., Seroogy, C.M., Wysocki, C.A., et al. (2019). FOXN1 compound heterozygous mutations cause selective thymic hypoplasia in humans. J. Clin. Invest. *129*, 4724–4738.

Gallo, V., Cirillo, E., Giardino, G., and Pignata, C. (2017). FOXN1 deficiency: from the discovery to novel therapeutic approaches. J. Clin. Immunol. *37*, 751–758.

Gong, J., Zhou, D., Jiang, L., Qiu, P., Milewicz, D.M., Chen, Y.E., and Yang, B. (2020). In vitro lineage-specific differentiation of vascular smooth muscle cells in response to SMAD3 deficiency: implications for SMAD3-related thoracic aortic aneurysm. Arterioscler. Thromb. Vasc. Biol. *40*, 1651–1663.

Gordeeva, O.F., and Nikonova, T.M. (2013). Development of experimental tumors formed by mouse and human embryonic stem and teratocarcinoma cells after subcutaneous and intraperitoneal transplantations into immunodeficient and immunocompetent mice. Cell Transplant. *22*, 1901–1914.

Grabowska, A.I., and Wilanowski, T. (2017). FOXN1 transcription factor in epithelial growth and wound healing. Mol. Cell. Biol *37*, e00110–17.

Hentze, H., Soong, P.L., Wang, S.T., Phillips, B.W., Putti, T.C., and Dunn, N.R. (2009). Teratoma formation by human embryonic stem cells: evaluation of essential parameters for future safety studies. Stem Cell Res. *2*, 198–210.

Lee, D., Prowse, D.M., and Brissette, J.L. (1999). Association between mouse nude gene expression and the initiation of epithelial terminal differentiation. Dev. Biol. *208*, 362–374.

Meier, N., Dear, T.N., and Boehm, T. (1999). Whn and mHa3 are components of the genetic hierarchy controlling hair follicle differentiation. Mech. Dev. *89*, 215–221.

Nowell, C.S., Bredenkamp, N., Tetelin, S., Jin, X., Tischner, C., Vaidya, H., Sheridan, J.M., Stenhouse, F.H., Heussen, R., Smith, A.J.H., et al. (2011). Foxn1 regulates lineage progression in cortical and medullary thymic epithelial cells but is dispensable for medullary sublineage divergence. PLoS Genet. *7*, e1002348.

Palamaro, L., Romano, R., Fusco, A., Giardino, G., Gallo, V., and Pignata, C. (2014). FOXN1 in organ development and human diseases. Int. Rev. Immunol. *33*, 83–93.

Pantelouris, E.M. (1973). Athymic development in the mouse. Differentiation *1*, 437–450.

Rolstad, B. (2001). The athymic nude rat: an animal experimental model to reveal novel aspects of innate immune responses? Immunol. Rev. *184*, 136–144.

Rota, I.A., and Dhalla, F. (2017). FOXN1 deficient nude severe combined immunodeficiency. Orphanet J. Rare Dis. *12*, 6.

Shayne, M.B., Song, J., Xu, J., and Patrick, A.L. (2019). Husbandry efforts for housing and maintaining immunodeficient transgenic rabbits. Lab. Anim. Sci. Prof. *7*, 58–59.

Song, J., Yang, D., Ruan, J., Zhang, J., Chen, Y.E., and Xu, J. (2017). Production of immunodeficient rabbits by multiplex embryo transfer and multiplex gene targeting. Sci. Rep. *7*, 12202.

Szadvari, I., Krizanova, O., and Babula, P. (2016). Athymic nude mice as an experimental model for cancer treatment. Physiol. Res. *65*, S441–S453.

Zhang, Z., Burnley, P., Coder, B., and Su, D.M. (2012). Insights on FoxN1 biological significance and usages of the "nude" mouse in studies of T-lymphopoiesis. Int. J. Biol. Sci. *8*, 1156–1167.

Zuklys, S., Handel, A., Zhanybekova, S., Govani, F., Keller, M., Maio, S., Mayer, C.E., Teh, H.Y., Hafen, K., Gallone, G., et al. (2016). Foxn1 regulates key target genes essential for T cell development in postnatal thymic epithelial cells. Nat. Immunol. *17*, 1206–1215.

Stem Cell Reports, Volume *16*

Supplemental Information

Development of the Nude Rabbit Model

Jun Song, Mark Hoenerhoff, Dongshan Yang, Ying Yang, Cheng Deng, Luan Wen, Linyuan Ma, Brooke Pallas, Changzhi Zhao, Yui Koike, Tomonari Koike, Patrick Lester, Bo Yang, Jifeng Zhang, Y. Eugene Chen, and Jie Xu

Title:

Development of the Nude Rabbit Model

Supplementary information

Supplementary Figure 1. Non-nude (WT and *FOXN1*$^{+/-}$) rabbits outgrew NuRabbits, beginning at eleven weeks of age. Data presented as mean $\pm$ standard error of means (SEM) from fifteen non-nude or eleven nude rabbits at each time point. Related to Figure 1.

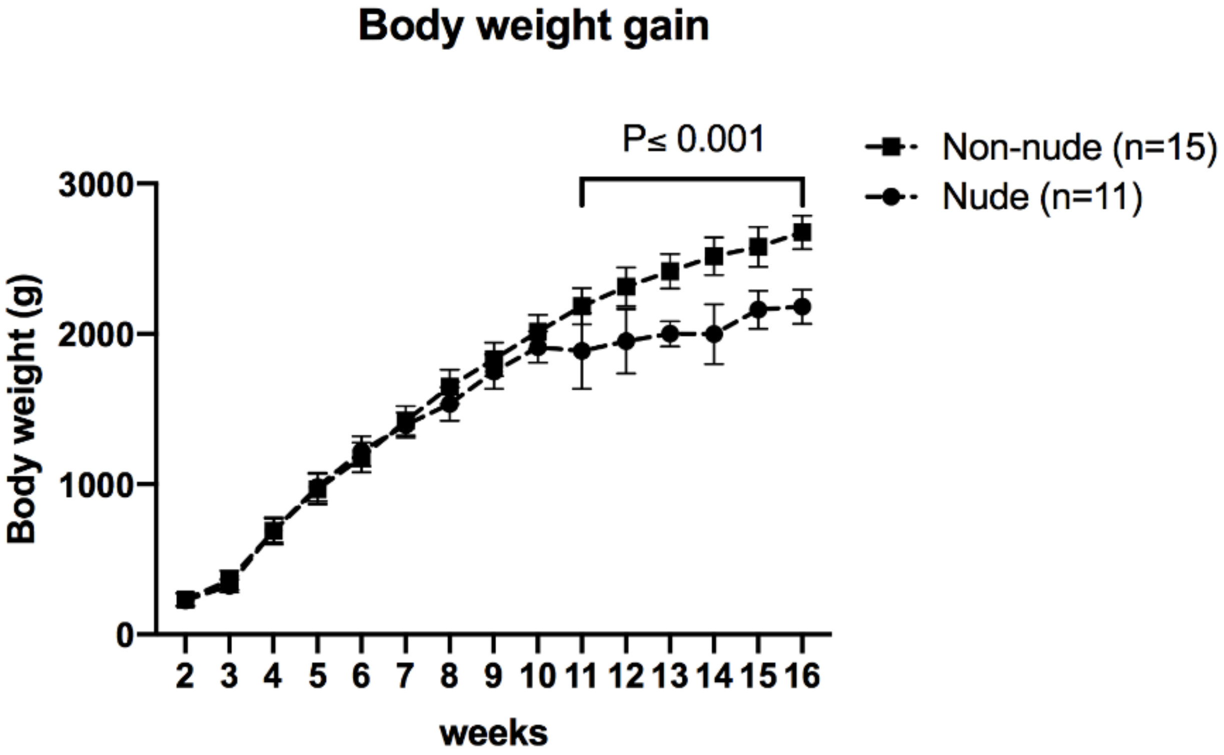

Supplementary Figure 2. Nail dystrophy in NuRabbits (right), compared to normal nail growth in a wild type rabbit (left). Related to Figure 2.

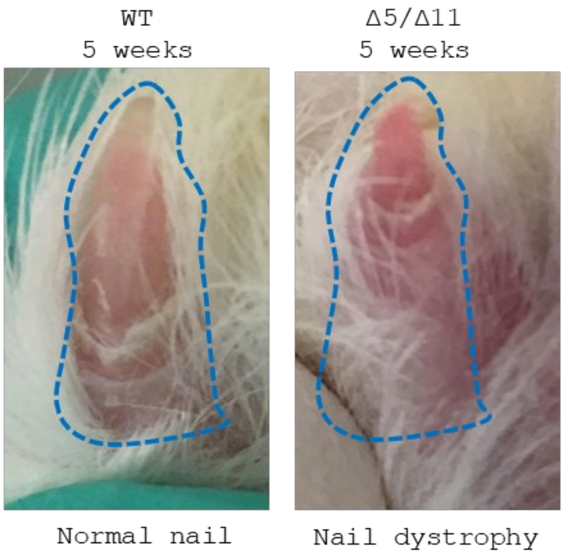

Supplementary Figure 3. Lung pathology of animal 17M049. NuRabbit 17M049 without prophylactic antibiotics developed severe bacterial bronchopneumonia. There is filling of bronchioles (arrowhead) and alveoli with neutrophils, and multifocal foci of necrosis (arrow) characterized by hypereosinophilic cellular debris and degenerative neutrophils scattered within the parenchyma. Scale bar: 50 µm. Related to Figure 3.

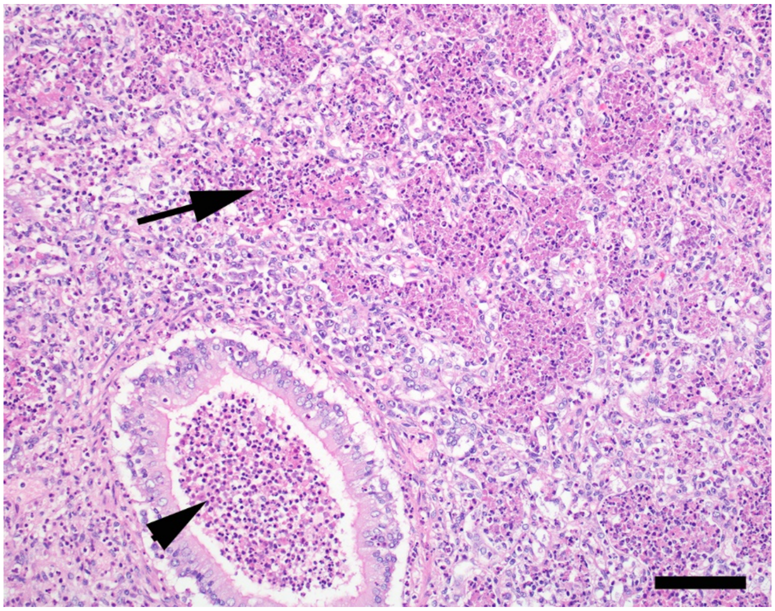

Supplementary Figure 4. Hair loss phenotype in a homozygous Δ10/Δ10 NuRabbit at different ages. Related to Figure 2.

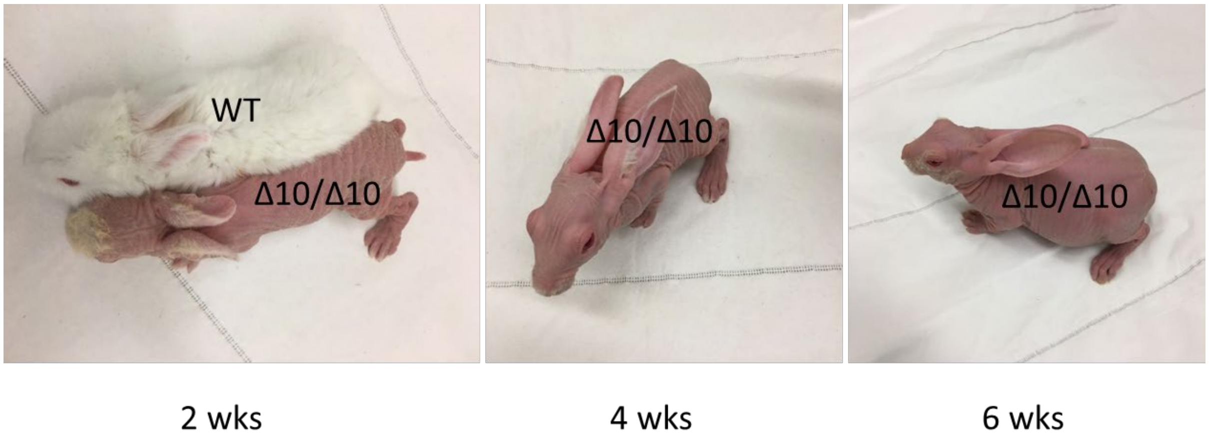

Supplementary Figure 5. Rabbit allows easy mammary gland intraductal injection. (A) Representative image of a rabbit nipple. (B) Visible mammary gland ducts after intraductal injection of PBS with 0.04% trypan blue using a 27 G needle in a rabbit. (C) The mouse nipple (within the white cycle) and a 27 G size needle. Related to Figure 4.

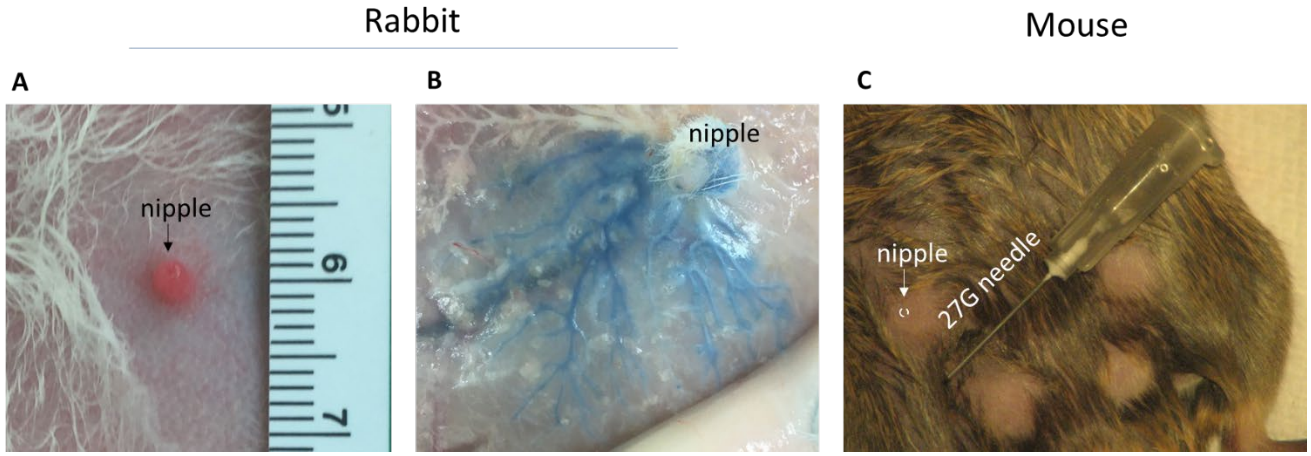

Supplementary Video 1. Restoration of blood flow in a NuRabbit after successful transplantation of a TEBV to its left common carotid artery. Related to Figure 5.
